# Analysis of Genome-Wide Copy Number Variations in Chinese Indigenous and Western Pig Breeds by 60 K SNP Genotyping Arrays

**DOI:** 10.1371/journal.pone.0106780

**Published:** 2014-09-08

**Authors:** Yanan Wang, Zhonglin Tang, Yaqi Sun, Hongyang Wang, Chao Wang, Shaobo Yu, Jing Liu, Yu Zhang, Bin Fan, Kui Li, Bang Liu

**Affiliations:** 1 Lab of Molecular Biology and Animal Breeding, Key Laboratory of Agricultural Animal Genetics, Breeding and Reproduction of Ministry of Education, Huazhong Agricultural University, Wuhan, PR China; 2 Key Laboratory of Farm Animal Genetic Resources and Germplasm Innovation of Ministry of Agriculture, Institute of Animal Science, Chinese Academy of Agricultural Sciences, Beijing, PR China; Wageningen UR Livestock Research, Netherlands

## Abstract

Copy number variations (CNVs) represent a substantial source of structural variants in mammals and contribute to both normal phenotypic variability and disease susceptibility. Although low-resolution CNV maps are produced in many domestic animals, and several reports have been published about the CNVs of porcine genome, the differences between Chinese and western pigs still remain to be elucidated. In this study, we used Porcine SNP60 BeadChip and PennCNV algorithm to perform a genome-wide CNV detection in 302 individuals from six Chinese indigenous breeds (Tongcheng, Laiwu, Luchuan, Bama, Wuzhishan and Ningxiang pigs), three western breeds (Yorkshire, Landrace and Duroc) and one hybrid (Tongcheng×Duroc). A total of 348 CNV Regions (CNVRs) across genome were identified, covering 150.49 Mb of the pig genome or 6.14% of the autosomal genome sequence. In these CNVRs, 213 CNVRs were found to exist only in the six Chinese indigenous breeds, and 60 CNVRs only in the three western breeds. The characters of CNVs in four Chinese normal size breeds (Luchuan, Tongcheng and Laiwu pigs) and two minipig breeds (Bama and Wuzhishan pigs) were also analyzed in this study. Functional annotation suggested that these CNVRs possess a great variety of molecular function and may play important roles in phenotypic and production traits between Chinese and western breeds. Our results are important complementary to the CNV map in pig genome, which provide new information about the diversity of Chinese and western pig breeds, and facilitate further research on porcine genome CNVs.

## Introduction

Copy number variations (CNVs) refer to the structurally genomic variations from hundreds of bases to several kilo-bases and the relevant complex mutations in the construction of chromosomes. Since the duplication of *Bar* gene in Drosophila melanogaster was first reported by Bridge to cause the Bar eye phenotype, more and more scientists focused on such DNA structural duplication [1]. In 2004, Iafrate firstly illustrated numerous structural variations in human genome, and then Redon *et al*. defined the copy number variations in human genome [2], [3].

Compared with the most frequent SNP marker, CNVs cover wider genomic regions and have potentially larger effects to change gene structure and dosage, exposing recessive alleles, and alternating gene regulation and other mechanisms. In humans, most studies on CNVs are shown to associate with Mendelian diseases and complex genetic disorders, such as major depressive disorder [4], schizophrenia [5], cancer [6], [7], body mass index [8], [9], and various congenital defects [10]. Besides disorders, CNVs are also important to maintain the normal phenotypic variability. Many studies were conducted on the influence of CNVs on the phenotype of domestic animals, such as copy number variation in intron 1 of *SOX5* leading to the Pea-comb phenotype in chickens [11], a 4.6-kb intronic duplication in *STX17* (Syntaxin 17) causing hair greying and melanoma in horse [12], [13], and the duplication of *FGF3*, *FGF4*, *FGF19* and *ORAOV1* resulting in hair ridge and predisposition to dermoid sinus in Ridgeback dogs [14]. A 110 kb microdeletion in the maternally imprinted *PEG3* domain was found, which results in a loss of paternal *MIMT1* expression and causes late term abortion and stillbirth in cattle [15], and ectopic *KIT* copy number variation may be associated with gonadal hypoplasia in Northern Finncattle and Swedish Mountain cattle [16]. George. Liu and his team detected CNVRs of cattle genomes by using different methods in diverse cattle breeds, and they also found evidence of CNVs relating with residual feed intake and resistance to gastrointestinal nematodes [17]–[21]. In pigs, only a few such studies are reported. For example, for the color of pig coat, the duplication and the exon-17-skipping mutation of *KIT* are responsible for the dominant white phenotype and peripheral blood cell [22]–[24].

Currently, CNVs can be identified using several technologies based on either ultra-dense genotyping with SNP chips, the hybridization of DNA in BAC/PAC/oligonu-cleotide arrays or high-throughput sequencing. The comparative genomic hybridization (CGH) based approach and high-throughput sequencing have excellent performance in refined resolution and relative signal intensities, while the SNP genotyping array has the advantage in both genome-wide association studies (GWAS) and CNV detection. The SNP arrays can collect normalized total signal intensity (Log R ratio-LRR) and allelic intensity ratios (B allele frequency-BAF) which represent overall copy numbers and allelic contrasts [25]. Besides, the SNP arrays need fewer samples than CGH arrays in an experiment, thus being more cost-effective and allowing users to increase the number of tested samples on a limited budget [26]. Nowadays, SNP arrays have been routinely used for CNV detection in humans and other organisms, and manufacturers of SNP genotyping arrays have incorporated non-polymorphic markers into their arrays to improve the coverage of SNP arrays for CNV analysis [27].

Since the accomplishment of the first human genome CNV map, many reports have been published on the characterization of the genomic architecture of CNVs in domestic species. Low-resolution CNV maps were produced for cattle, dog, pig, goat, sheep, chicken, duck, turkey and horse, showing that these structural polymorphisms comprise a significant part of these genomes [17], [28]–[32]. Based on porcine SNP60 Beadchip and aCGH, Fadista *et al*. [33], Ramayo-Caldas *et al*. [29], Wang *et al*. [34], [35], Chen *et al*. [36], and Li *et al*. [37] have identified a large amount of CNVs in pig genome among different breeds, including several Chinese breeds.

Chinese indigenous breeds have larger genetic diversity than European breeds, leading to the tremendous phenotypic differences among them. In the present study, we analyzed the difference in CNVs between Chinese indigenous breeds and western breeds by using Porcine SNP60 BeadChip and PennCNV algorithm, and performed a genome-wide CNV detection in 302 pigs from six Chinese indigenous breeds, three European breeds and one hybrid. This study produced a comprehensive map of CNVs in the pig genome, which could give new insight to the interspecific diversity of different breeds and facilitate further research on porcine genome CNVs.

## Materials and Methods

### Ethics Statement

The whole blood samples were collected in strict accordance with the protocol approved by the Biological Studies Animal Care and Use Committee of Hubei Province, PR China. All efforts were made to minimize any discomfort during blood collection.

### Animal samples

The animals were composed of 302 pigs from nine pure breeds and one hybrid, including six Chinese indigenous breeds (45 Tongcheng pigs-TC, 23 Laiwu pigs-LW, 40 Luchuan pigs-LC, 23 Bama pigs-BM, 26 Wuzhishan pigs-WZS, 24 Ningxiang pigs-NX) and three western breeds (33 Yorkshire pigs-YS, 33 Landrace pigs-LD, 32 Duroc pigs-Dur) and one hybrid (23 Tongcheng × Duroc crossbred pigs-BC).

Genomic DNA samples were extracted from whole blood of all pigs using a standard phenol/chloroform method. All DNA samples were analyzed by spectrophotometry and agarose gel electrophoresis.

### SNP array genotyping and quality control

All 302 pigs were genotyped with the Porcine SNP60 Genotyping BeadChip (Illumina Inc., USA) using the Infinium II Multisample assay (Illumina Inc.). SNP arrays were scanned using iScan (Illumina Inc.) and analyzed using GenomeStudio (Version 3.2.2, Illumina, Inc.).

In order to exclude poor-quality DNA samples and decrease potential false-positive CNVs, only the samples at a call rate >98% and call frequency >90% were reserved. After quality control, 286 of the 302 samples were retained for CNV detection (43 Tongcheng pigs, 22 Laiwu pigs, 39 Luchuan pigs, 21 Bama pigs, 23 Wuzhishan pigs, 23 Ningxiang pigs, 31 Yorkshire pigs, 31 Landrace pigs, 30 Duroc pigs and 23 Tongcheng ×Duroc crossbred pigs). For subsequent data analysis, a subset of 52,089 SNPs was selected by removing the SNPs located in sex chromosomes and those not mapped in the Sscrofa10.2 assembly.

### Identification of pig CNVs

PennCNV was used for CNV identification by integrating a Hidden Markov Model (HMM) for high resolution copy number variation detection with whole-genome SNP genotyping data [38]. This algorithm incorporates multiple sources of information, including total signal intensity data of log R Ratio (LRR) and B allele frequency (BAF) at each SNP marker, the distance between neighboring SNPs, the population frequency of B allele (PFB) of SNPs, and the pedigree information where available. The LRR and BAF were exported using Illumina BeadStudio software. There were three arguments in PennCNV including -test, -trio and -joint. Individual-based CNV calling was performed using the -test with default parameters of the HMM model by integrating Log R Ratio, BAF, population allele frequency and the SNP distance. To reduce the false discovery rate in CNV calling, we adopted the calling criteria that the standard deviation (SD) of LRR was under or less than 0.35, and the CNV contained three or more consecutive SNPs. All putative CNVs identified in this study were pooled across breeds. Finally the CNV regions (CNVRs) were determined by aggregating the overlapping CNVs identified across all samples according to the previously published protocols [3].

### Gene contents and functional annotation

Gene contents in the identified CNVRs were retrieved from the Ensembl Genes 70 Database using the BioMart (http://asia.ensembl.org/biomart/martview/) data management system. Functional annotation of these genes was performed with the DAVID bioinformatics resources 6.7 (http://david.abcc.ncifcrf.gov/) for Gene Ontology (GO) terms and Kyoto Encyclopedia of Genes and Genomes (KEGG) pathway analysis. Considering the limited number of genes in the pig genome have been annotated, we first converted the pig Ensembl gene IDs to homologous human Ensembl gene IDs by BioMart, and then carried out the GO and pathway analysis. Statistical significance was assessed by using *P* value of a modified Fisher's exact test and Benjamini correction for multiple testing.

### Validation of CNVRs by qPCR

CNVRs were confirmed by qPCR using the Roche LightCyclerW 480 Detection System and the 2^−ΔΔCt^ method which compares the ΔCt (cycle threshold (Ct) of the target region minus Ct of the control region) value of samples with CNV to the ΔCt of a calibrator without CNV [39]. The primers were designed using the Primer Premier 5 software (Table S10 in File S1). As previously reported, the copy number of each CNVR was normalized against the *GCG* gene, a control region in the genome with no variation in the copy numbers between the pigs [29]. Triplicate wells of reactions (10 µL) contained 5 µL SYBR Green Real-time PCR Master Mix, 1 µL of 50 ng/µL gDNA, 0.3 µL 10 µM of each primer and 3.4 µL ddH_2_O. The cycling conditions consisted of 95°C for 10 min, 40 cycles (at 94°C for 30 sec, 60°C for 30 sec, and 72°C for 10 sec), and fluorescence acquisition at 72°C in single mode. The specific PCR products were confirmed by the results of melting curve analysis and agarose gel electrophoresis.

## Results and Discussion

### Genome-wide CNVs detection

**Figure 1:**
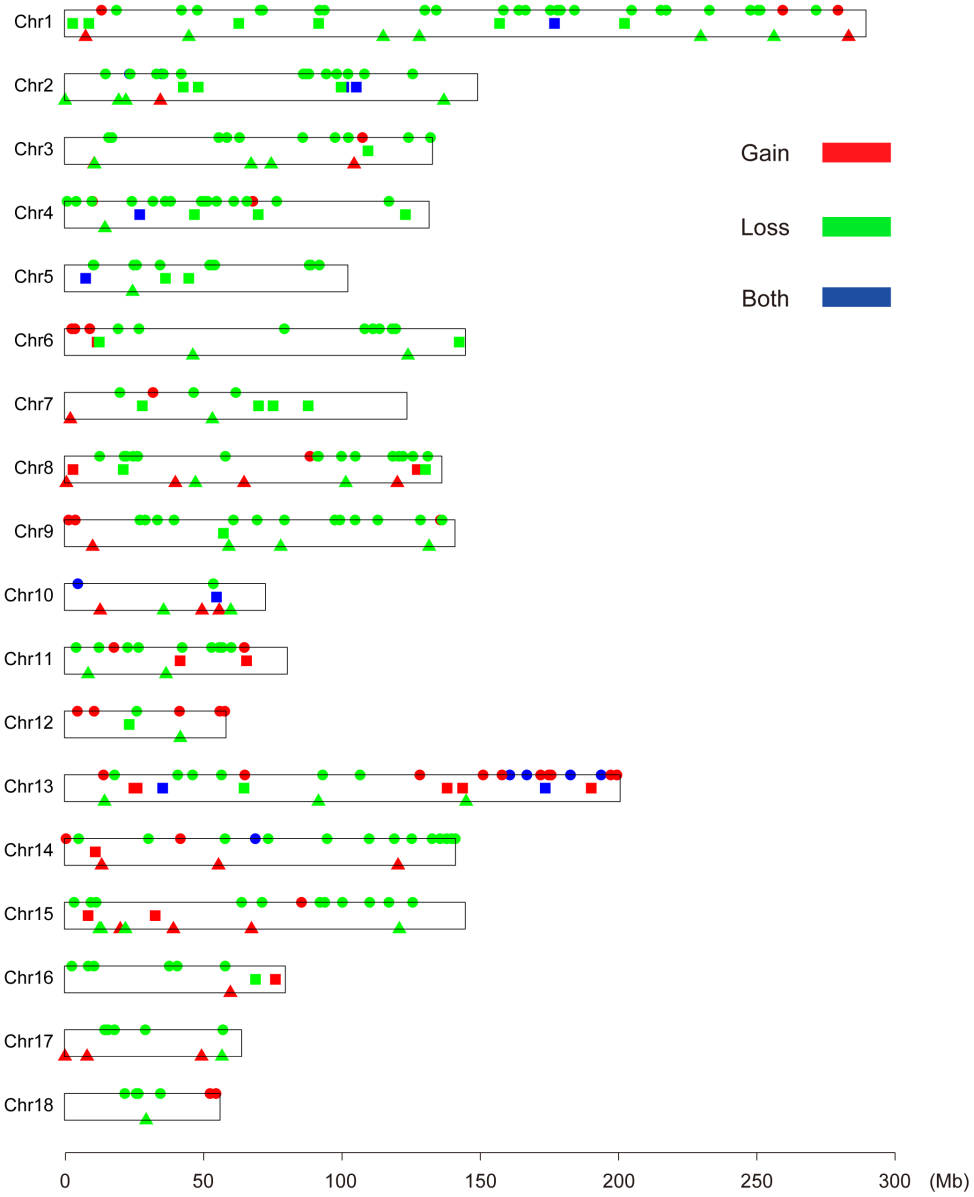
Genomic distribution of CNVRs in 18 pairs of autosomal chromosomes of pigs. The chromosomal locations of 348 CNVRs are indicated by lines. Y-axis values are chromosome names, and X-axis values are chromosome positions in Mb, which are proportional to the real size of swine genome sequence assembly (10.2). Round represents CNVRs identified only in Chinese indigenous breeds; triangle represents CNVRs identified only in western breeds; and quadrate represents those identified both in Chinese and western breeds.

A total of 1272 CNVs were assessed by PennCNV on 18 pairs of autosomal chromosomes and 348 CNVRs were acquired by aggregating overlapping CNVs (Table S1a in File S1), covering 150.49 Mb of the pig genome and about 6.14% of the autosomal genome sequence, with an average number of 4.45 CNVs per individual. Among the 348 CNVR events, 88 were found to be gain, 243, loss and 17, both (loss and gain within the same region) events, which were dispersed all over the 18 autosomes and ranged from 4.93 Kb to 12.41 Mb in length with a mean of 443.24 Kb and median of 170.77 Kb. The location and characteristics of all CNVRs on autosomal chromosomes are presented in Figure 1, showing that these CNVRs are not uniformly distributed among different chromosomes. The proportion of CNVRs on the 18 pairs of autosomal chromosomes varies from 1.17–20.91% and chromosome 1 harbors the greatest number (43) of CNVRs (Table 1).

**Table 1:** Chromosome distribution of CNVRs in pigs.

Chr	No. of CNVRs	No. of genes	Length of CNVRs (bp)	Length of chromosomes (bp)	Percentage (%)
1	43	73	23217110	315321322	7.36
2	26	59	11921008	162569375	7.33
3	18	23	2788734	144787322	1.93
4	22	51	8591089	143465943	5.99
5	18	35	5889799	111506441	5.28
6	16	45	3648860	157765593	2.31
7	12	40	2619625	134764511	1.94
8	28	20	8873483	148491826	5.98
9	22	53	5312989	153670197	3.46
10	10	14	1565092	79102373	1.98
11	17	20	5477336	87690581	6.25
12	8	15	741280	63588571	1.17
13	32	230	45721621	218635234	20.91
14	23	59	11437761	153851969	7.43
15	23	18	5627033	157681621	3.57
16	11	18	2673814	86898991	3.08
17	12	17	2291744	69701581	3.29
18	7	8	2087925	61220071	3.41
Total	348	798	150486303	2450713522	6.14

Excluding the CNVR event detected only in one individual, we obtained 166 CNVRs, with 107 loss, 42 gain and 17 both events. These CNVRs covered 103.01 Mb, and ranged from 5.75 Kb to 12.41 Mb with a mean of 620.57 Kb and median of 217.76 Kb (Table S1b in File S1). Previous studies always analyzed their results after eliminating the only individual event, but when compared with their results, more than 36% of the only individual events in our results overlapped with their data, suggesting the loss of much useful information by eliminating the only individual events. Thus, we analyzed the whole 348 CNVRs during the following research.

In our study, the 6.14% CNVR coverage in the autosomal genome sequence was consistent with the 0.31% to 5.84% coverage of analyzed genome on pigs reported previously [29], [34], [36]. In humans, CNVR coverage was reported to be as high as 12% of the genome when Redon *et al* first identified 1447 CNVRs in human genome [3]. The CNVs were anticipated to cover up to 13% of the human genome [40]. In bovines, the CNVR coverage was reported to be from 0.68% to 4.6% of the genome [17], [41], [42].

A great difference was found in the CNVR numbers among the nine breeds. Among the six Chinese indigenous breeds, the maximum number of CNVRs was detected in Luchuan pigs, accounting for 125 CNVRs (33.78%), followed by Tongcheng 84(22.7%) and Laiwu 76 (20.54%) pigs. The minimum number of CNVRs was only 35 (9.46%) in Wuzhishan pigs. With respect to European breeds, 54 (14.59%), 48 (12.97%) and 37 (10%) CNVRs were found in Yorkshire, Landrace and Duroc pigs, respectively. Altogether 240 unique CNVRs were detectable in the nine breeds, with Luchuan pigs harboring the maximum number (Table 2).

**Table 2:** Sample size and CNVs number detected in nine breeds.

Breed	sample size	CNVs number	CNVs per sample	CNVRs number	unique CNVRs	Frequency (%)
Tongcheng	43	194	4.51	84	33	22.70
Laiwu	22	160	7.27	76	41	20.54
Luchuan	39	279	7.15	125	64	33.78
Bama	21	90	4.29	36	6	9.73
Wuzhishan	23	67	2.91	35	15	9.46
Ningxiang	23	120	5.22	61	25	16.49
Yorkshire	31	113	3.65	54	25	14.59
Landrace	31	107	3.45	48	19	12.97
Duroc	30	79	2.63	37	12	10.00

The number of CNVs among individuals was variable. 39 individuals were only found one CNV event, 49 individuals were found two CNV events, and most individuals were three CNV events (Table S2 in File S1). With an increase in copy number variation, the detected individuals became fewer and fewer, indicating that most animals can survive through only a few CNVs. CNV numbers differed greatly among different pig populations too. The average number of CNVs per population was 127.2, ranging from 67 (Wuzhishan) to 279 (Luchuan). The maximum number of CNVs per sample was detected in Laiwu pigs (7.32 CNVs per sample on average) against the minimum number of 2.63 CNVs per animal in Duroc pigs. Similar to the finding in human [43], most CNVRs (68.77%) were restricted to one population, probably due to sampling variances or recent evolution events.

### Gene content of pig CNVRs

Totally, 798 genes within the identified CNVRs were retrieved from the Ensembl Genes 70 Database using the BioMart data management system, including 651 protein-coding genes, 10 pseudogenes, 30 miRNA, 49 snRNA, 35 snoRNA, 13 miscRNA, 7 rRNA, and 3 processed-transcripts. In CNVRs, 455 of the 798 genes were identified to be loss events, 303 and 39 genes, gain and both events, respectively (Table S3 in File S1). The average number of genes per Mb of 348 CNVRs was 5.29, which was less than that on the whole genome(8.62) according to the *Sus crofa* 10.2 assembly in Ensembl (http://a sia.ensembl.org/), suggesting that CNVs are located preferably in gene-poor regions, probably because changes in copy number for genes that perform essential functions are subject to strong purifying selection [44], [45].

In order to provide insight into the functional enrichment of the CNVs, Gene Ontology (GO) and Kyoto Encyclopedia of Genes and Genomes (KEGG) pathway analyses were performed with the DAVID bioinformatics resources. The Gene Ontology (GO) analysis revealed that CNV genes mainly participated in cell adhesion, phosphorus metabolic process, cell projection, phosphorylation, cellular component morphogenesis, cell differentiation, muscle cell development and other basic metabolic processes. The KEGG pathway analysis indicated that genes in CNVRs were involved in seven pathways including phosphatidylinositol signaling system, inositol phosphate metabolism, oocyte meiosis, cell cycle, leukocyte transendothelial migration, Alzheimer's disease and Huntington's disease (Table S4 in File S1 and S5 in File S1).

Additionally, 3258 QTLs out of 8000 which affect a wide range of traits, such as immune capacity, disease resistance, meat quality, growth and litter size, were found in 240 CNVRs by comparing the overlapping of CNVRs with QTLs in the pig QTLdb (http://www.animalgenome.org/cgi-bin/QTLdb/SS/index) (Table S6 in File S1).

### Differences between Chinese normal size pig and minipig breeds

In our population, four of the six Chinese breeds are normal size (Tongcheng, Laiwu, Luchuan and Ningxiang), the other two are minipig breeds. There are many differences of CNVs between these two type pigs. The four normal size breeds harbored 179 CNVRs, covering 80.59 Mb of pig genome sequence and 366 ensemble genes (table S7a and 7b in File S1). These genes mainly participate in cell adhesion, regulation of cell cycle, detection of stimulus, phosphate metabolic process, ATP biosynthetic process, muscle cell differentiation, purine nucleotide metabolic process, and regulation of growth. Many CNV-associate genes in these regions appear to be certain gene clusters or families, such as ubiquitin-conjugating enzyme family (*UBE2B*, *UBE2G2*), ATPase family (*ATP2A1*, *ATP13A5*, *ATP5J*), trefoil factor family (*TFF1*.*TFF2*, *TFF3*), and claudin family (*CLDN8*, *CLDN17*). These genes mainly involved in immune system and some human diseases [46]–[53]. *VCAN* (versican), *ADAM17* (ADAM metallopeptidase domain 17), *ITGB1BP1* (integrin beta 1 binding protein 1), *CDH19*, *ITGAD* and *PCDH15* were also playing important role in inflammation and other diseases [54]–[59]. Moreover, *CDH19* was evidenced as a copy number alterations (CNAs) target gene to impact central nervous system. *ZWINT* (ZW10 interacting protein PIK3C3), *PIK3C3* (phosphoinositide-3-kinase class 3) and *PTGS2* (prostaglandin-endoperoxide synthase 2) were important for cell proliferation [60]–[62], and *PIK3C3* and *PTGS2* were proved as candidate marker for production and reproductive traits in pigs [63]–[65]. These findings indicated that CNVs may have potential effect on immune response, production and reproductive traits of these pigs.

Bama pigs and Wuzhishan pigs are two famous Chinese minipig breeds, whose body weight are less than one third of modern commercial breeds. 21 CNVRs that only detected in these two breeds were picked out to investigate whether CNVs were contribute to their phenotypes. 49 ensemble genes were retrieved overlapped with these CNVRs, including 42 protein-coding genes, 4 snoRNAs, 2 snRNAs and 1 pseudogene (Table S7c and 7d in File S1). Among these genes, *STX17* was evidenced that a 4.6-kb intronic duplication of it would cause hair greying and melanoma in horse [12]; *INPP5A* (inositol polyphosphate-5-phosphatase), *TCERG1L* (transcription elongation regulator 1-like), *FOXL1* (forkhead box L1) and *POLR1D* (polymerase (RNA) I polypeptide D) mainly participated in human cancer and some other diseases [66]–[69]. Some genes such as *MTCH2* (mitochondrial carrier 2), *BNIP3* (BCL2/adenovirus E1B 19 kDa interacting protein 3) and *DPYSL4* (dihydropyrimidinase-like 4) were playing essential role in cell apoptosis [70]–[72], some as *CDK13* (cyclin-dependent kinase 13) and *SGSM1* (small G protein signaling modulator 1) participated in regulation of cell circle, differentiation and proliferation [73], [74]. According to the function of CNV-associated genes in the six breeds, we assumed that CNVs may contribute to disease resistance and stress resistance of all these breeds, but unfortunately, we didn't find sound evidence for CNVs impacting the growth of these two minipig breeds.

An average of 6.04 CNVs was obtained for each sample in the four Chinese normal size breeds, while only 3.6 was in the two minipig populations (table 2). This means more variations occurred in four nomal size breeds than minipigs. Despite the population size of these breeds, the most convincing reason for such small number of CNVs in minipigs is domestic methods and artificial selection. These two breeds were both raised in mountain area, which means highly inbreeding was inevitable because of terrible traffic condition, leading to less variation in these breeds. After long period of natural and artificial selection, these two breeds became more and more conservative and steady.

### Differences of CNVRs in Chinese indigenous breeds and western breeds

A total of 213 CNVRs were identified to exist only in the six Chinese native breed populations, 60, in three western breeds and 49, in all. Fewer CNVRs were detected in western breeds, with only one CNVR detected on chromosomes 4, 5, 12 and 18 (Figure 1, Table S8a in File S1). Two potential reasons may explain such a small number of CNVRs. Firstly, the population size of the western breeds of pigs in this study is smaller than that of Chinese native breeds. Secondly, strongly artificial selection of the western commercial breeds tended to make them purified, thus decreasing variations in the population. However, Chinese native pigs were subjected to lower selection intensities and had fewer selection signatures, which may conserve most variations after evolution.

A total of 109 ensemble genes overlapped with CNVRs of western breeds, including 99 protein-coding genes, 2 pseudogenes, 1 miRNA, 5 snRNA and 2 snoRNA. GO analysis revealed that these genes were mainly involved in cell adhesion, regulation of phosphorylation and cell proliferation. Differ from western breeds, 499 ensemble genes were retrieved overlapping CNVRs of Chinese indigenous breeds, including 389 protein-coding genes, 6 pseudogenes, 26 miRNA, 35 snRNA, 28 snoRNA, 8 miscRNA, 5 rRNA, and 2 processed-transcripts. By GO analysis, we found that these genes were involved in not only cell adhesion and regulation of phosphorylation, but also behavior, neuron differentiation and regulation of T cell receptor signaling pathway (Table S8b in File S1). For further pathway analysis, we found Chinese breeds specific CNV-associated genes such as *ITGB2*, *CLDN8/14/17*, *CDK13*, *JAM2*, *ALCAM* and *VCAN* played important role in immune system, Leukocyte transendothelial migration, T cell receptor signaling pathway and other pathways. Some of the important genes were mentioned in proceeding part. These results illustrated that CNVs may play important role in immune system among Chinese breeds and growth among western breeds. As we all know that Chinese indigenous breeds and western breeds show obvious differences in many aspects, our results may provide a genetic explanation for the difference in disease resistance and growth rate.

### Comparison with previous reports

Our results were compared with previous reports on porcine genomic CNVs (Table S9 in File S1). The first research on CNVs of pig genome by using Porcine SNP60 Beadchip was reported by Ramayo-Caldas [29], who detected 49 CNVRs from 55 pigs of Iberian x Landrace cross. Thirty out of the 49 CNVRs were overlapped with our results. Wang *et al*. detected 382 CNVRs based on the Porcine SNP 60 genotyping data of 474 individuals from three pure breed populations and one Duroc × Erhualian crossbred population [34]. Among the 382 CNVRs, 97 regions were found to overlap with our results. Using the same method, Chen *et al.* detected 565 CNVRs in 1693 pigs from 18 diverse populations, and 179 of them were overlapped with our results. Using 720 K array CGH, Li *et al.* identified 259 CNVRs in 12 animals including three Chinese native pigs, 5 European pigs, 2 synthetic pigs and 2 crossbred pigs (Landrace × DIV pigs) [37], and only 15 regions were found to be identical or overlapped with our data. Only 3 regions were found in all reports, containing 9 genes, with 7 of them being protein coding genes (table S9e in File S1).

The potential reasons for the differences between our results and other studies are as follows. First, there was a difference in population size and genetic background between our study and others. In our study, the CNVs of the four Chinese indigenous breeds including Luchuan, Laiwu, Ningxiang and Wuzhishan pigs were reported for the first time, involving lots of new breed-specific regions. Second, platforms (SNP genotyping array and CGH array) and calling algorithms, which are different in the calling technique, resolution and genome coverage, might contribute to the discrepancy of the CNVs detected. Third, our results were based on genome assembly *sus scrofa* 10.2, and except for Chen's research, the aforementioned reports were all based on genome assembly *sus scrofa* 9.0 version. As our data needed to be converted from 10.2 to 9.0, great differences might arise during the transformation, causing the deviation between our results and others. This is also occurred in CNV studies of other mammals [17], [75], [76].

### Validation of CNVR by real-time quantitative PCR (qPCR)

**Figure 2:**
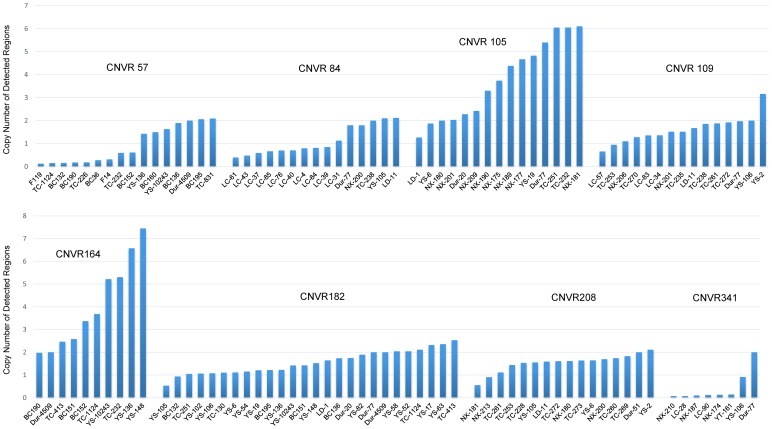
QPCR validation of 8 identified CNVRs. The x-axis represents the animals and the y-axis shows the relative quantification value.

Quantitative real time PCR (qPCR) was used to validate 12 CNVRs chosen from the 348 CNVRs detected in the study. These 12 CNVRs represent different predicted status of copy numbers (i.e., loss, gain and both) and different CNVR frequencies. 9 (75%) of them were in agreement with the prediction by PennCNV (Figure 2).

Except for CNVR187, 11 CNVRs contained important functional genes. The CNVR164 contained Mast/stem cell growth factor receptor gene, also known as *KIT* gene. *KIT* gene was considered to affect colors and their distribution in pigs with a 450 kb long duplication and the exon-17-skipping mutation. The copy number of *KIT* in dominant white color was higher than any other patterns [77], [78]. In our data, the copy numbers of three detected Yorkshire pigs were from 4 to 6, while the other detected samples including Tongcheng (two-black-end) and Duroc pigs (red) had approximately 2 copies. This result is consistent with the previous reports and prediction of PennCNV analysis based on SNPs chip. The CNVR182 locus contained *MAPK10* gene, which is a member of mitogenactivated protein kinase (MAPK) superfamily, and plays an important role in cancer and some other diseases [79]. The *MAPK10* is also associated with Hirschsprung disease as a candidate CNV gene [80]. In our data, the copy numbers of 11 samples out of 26 were identified as loss events, corresponding with PennCNV prediction. There has been no report so far about the function of *MAPK*10 in pigs, and our results indicate that *MAPK*10 may play an important role in pigs. Another 7 genes were detected in our validated CNVRs, such as *VCAN*, *CIB4*, *VCPIP1*, *ALG14*, *FAM5C*, *ZFPM2* and *DOK5*. Copy number variations were identified in all these seven genes, and these variations may affect their function in immunity, development, and growth.

## Conclusions

In this study, we described a map of porcine CNVRs between six Chinese indigenous breeds and three western breeds based on Porcine SNP60 genotyping data of 302 pigs. The results revealed that 213 CNVRs belong to the Chinese native breed populations, and 60 CNVRs, to the western breeds. We also discussed the CNV characters of four Chinese normal size breeds (Luchuan, Tongcheng and Laiwu pigs) and two minipig breeds (Bama and Wuzhishan pigs). Functional annotation suggested that these CNVRs and CNV-associate genes are involved in variety of molecular function and may play important roles in phenotypic and production traits difference between Chinese and western pig breeds.

## Supporting Information

File S1 **Supporting tables. Table S1, Information of identified CNVRs.** The description and information of all 348 identified CNVRs were showed in Table S1a. The information of 166 CNVRs which were identified by excluding the CNVR event detected only in one individual were showed in Table S1b. **Table S2, The CNVs numbers detected in 286 individuals.** **Table S3, Information of genes in the identified CNVRs. Table S4, Gene ontology (GO) analysis of genes in identified CNVRs. Table S5, Pathway analysis of genes in identified CNVRs. Table S6, Previously reported QTLs overlapping with identified CNVRs. Table S7, Information of CNVRs in Chinese normal size and minipig breeds.** Table S7a showed the information of CNVRs in four Chinese normal size pig breeds. Table S7b showed the information of genes in the CNVRs of four Chinese normal size pig breeds. Table S7c showed the information of CNVRs in two Chinese minipig breeds: Bama and Wuzhishan pigs. Table S7d showed the information of genes in the CNVRs of Bama and Wuzhishan pigs. **Table S8, Information of CNVRs and CNV-involved genes in Chinese indigenous and western breeds.** Table S8a showed information of CNVRs in Chinese indigenous and western breeds. Table S8b showed information of genes in the CNVRs of Chinese indigenous and western breeds. **Table S9, Comparison between identified CNVRs and those reported in other pig CNVR papers. Table S10, Information and the primers used in qPCR analysis of the 12 CNVRs chosen to be validated.**
(XLSX)
